# Editorial: Interactions among immune cells in leishmaniasis: exploring markers, enzymes and cytokines

**DOI:** 10.3389/fimmu.2026.1847070

**Published:** 2026-05-04

**Authors:** Herbert Leonel de Matos Guedes, Daniel Claudio Oliveira Gomes, Ramona Hurdayal

**Affiliations:** 1 Universidade Federa do Rio de Janeiro, Laboratório de Imunobiotecnologia, Instituto de Microbiologia Paulo de Góes, Rio de Janeiro, Brazil; 2 Fundação Oswaldo Cruz, Instituto Oswaldo Cruz, Laboratório de Imunologia Clínica, Rio de Janeiro, Brazil; 3 Universidade Federal do Espírito Santo, Núcleo de Doenças Infecciosas, Vitória, Espírito Santo, Brazil; 4 University of Cape Town, Department of Molecular and Cell Biology, Cape Town, South Africa

**Keywords:** clinical management, host-parasite interaction, immunometabolism, innate & adaptive immunity, leishmaniasis, therapeutics

Leishmaniasis remains a global health challenge, affecting millions of individuals across endemic regions worldwide. Although substantial research has advanced our understanding of host-parasite interactions, important gaps persist in defining the cellular and molecular networks that determine disease outcomes. This Research Topic,*“Interactions Among Immune Cells in Leishmaniasis: Exploring Markers, Enzymes and Cytokines,”* brings together eleven studies that collectively illuminate the immunological complexity of leishmaniasis, offering integrative perspectives spanning fundamental immunology, genetics, immunometabolism, therapeutic innovation, tissue microenvironments, and clinical management.

A central theme emerging from these contributions is the delicate balance between regulatory and effector T cell responses. Kamran et al. demonstrate that patients with active visceral leishmaniasis exhibit expanded CD4^+^CD127^-/low^CD25^high^ regulatory T cells capable of suppressing protective effector responses, and that normalization of these subpopulations following treatment defines a regulatory signature associated with disease pathogenesis. This raises the possibility that targeted Treg modulation could enhance therapeutic efficacy. Extending this concept of T cell dysfunction, Covre et al. reveal that lesional CD4^+^ T cells can acquire senescent and cytotoxic features, contributing to tissue pathology through bystander cytolysis. Together, these findings demonstrate that impaired T cell immunity in leishmaniasis encompasses not only regulatory suppression but also age-associated senescence and aberrant cytotoxicity, broadening our understanding of adaptive immune dysregulation.

Genetic background further shapes immune responses in ways that challenge longstanding paradigms. Sohrabi et al. provide a compelling example of epistasis by showing that combining genes from two resistant mouse strains paradoxically yields susceptibility. The newly generated B10.O20 strain developed large cutaneous lesions, high parasite burdens, intense CD11b^+^Gr1^+^ and CD11b^+^CD193^+^ cell infiltration, and mixed cytokine responses, whereas the parental O20 strain exhibited resistance despite lacking classical Th1-associated cytokine production or nitric oxide release. These observations highlight that resistance mechanisms extend beyond the conventional Th1/Th2 dichotomy and underscore the importance of incorporating broader genetic models to better approximate the diversity of human immune responses.

Tissue context also profoundly influences disease expression. Da-Rocha et al. establish an important murine model of mucosal leishmaniasis by infecting BALB/c mice with *Leishmania amazonensis* in nasal tissues. In contrast to dermal infection, mucosal infection resulted in higher parasite loads, pronounced tissue destruction, elevated cytotoxic and pro-inflammatory mediators, and reduced IL-10^+^ CD4^+^ T cell responses. This skewed inflammatory-regulatory imbalance closely mirrors the pathology observed in human mucocutaneous disease and provides a valuable experimental platform for investigating mechanisms underlying this severe clinical manifestation, which has long lacked adequate preclinical models.

Beyond cellular composition and genetic determinants, immunometabolism emerges as a critical regulator of host-parasite interactions. Aruleba et al. bridge metabolic and immunological analysis through metabolomic profiling of sodium stibogluconate delivered in a non-ionic surfactant vesicular formulation in experimental visceral leishmaniasis. Their findings demonstrate that effective therapy not only reduces parasite burden but also modulates disrupted metabolic pathways and shifts the host metabolome in ways consistent with parasite clearance and immune activation. Early infection markers such as valine, lactic acid, and glycerol-1-oleate, along with treatment-associated changes such as elevated glycine and reduced ribitol, suggest that metabolic signatures could serve as non-invasive biomarkers for disease monitoring and therapeutic response. These data reinforce the concept that metabolic reprogramming is both a consequence and driver of immune dysfunction.

Macrophage biology remains central to the immunopathology of leishmaniasis, and several studies deepen this understanding. Fry et al. demonstrate that HIF-α signaling regulates macrophage inflammatory responses during *Leishmania major* infection. Notably, HIF-α-dependent transcriptomic changes were driven predominantly by the inflammatory microenvironment rather than direct parasite sensing, leading to suppression of protein translation pathways under pro-inflammatory conditions. This work identifies HIF-α as a key immunoregulatory node and highlights the limitations of simplistic M1/M2 polarization models. When considered alongside metabolic reprogramming (Aruleba et al.) and adaptive immune regulation (Kamran et al.; Covre et al.), these findings emphasize the interconnected nature of anti-leishmanial immunity.

Innate immunity, particularly neutrophil dynamics, also plays a decisive role during early infection. Ihedioha et al. show that *Leishmania major* enhances neutrophil migration and survival via DKK1-LRP6 signaling at infection sites. Disruption of parasite surface components impaired sustained neutrophil recruitment, revealing a parasite-driven mechanism that exploits neutrophils as both initial responders and potential reservoirs for dissemination. Targeting the DKK1-LRP6 axis may therefore offer new therapeutic strategies to rebalance early inflammatory responses toward protective immunity. Therapeutic innovation is further represented by the study of Pimenta et al., who demonstrate that blue LED phototherapy exerts significant anti-leishmanial effects both *in vitro* and in murine models of tegumentary disease. This non-pharmacological intervention, characterized by low toxicity and minimal risk of resistance, may represent a promising adjunct or alternative therapy, particularly in resource-limited settings where conventional treatments remain inaccessible or poorly tolerated.

Clinical perspectives enrich this Research Topic by illustrating how immune modulation influences disease presentation. Shahini et al. report a disseminated *Leishmania* infection in a patient with rheumatoid arthritis undergoing prolonged immunosuppressive therapy, underscoring the diagnostic complexity posed by overlapping inflammatory and infectious manifestations. Their report highlights the importance of vigilant screening for opportunistic infections in immunocompromised individuals living in or traveling from endemic regions. Complementing this, Vellozo et al. review macrophage plasticity as a determinant of host outcome in *Leishmania* and *Trypanosoma cruzi* infections, exploring host-directed strategies in the context of M1 and M2 pathways. They emphasize both the promise and challenges of therapeutically manipulating macrophage polarization in parasitic diseases of global significance.

Taken together, these contributions highlight several guiding principles. Disease outcomes in leishmaniasis arise from coordinated interactions among immune populations rather than isolated cellular defects. Regulatory T cells, senescent effectors, macrophages, neutrophils, and dendritic cells operate within a dynamic network in which perturbations in one compartment influence the broader response. Host genetic background further modulates these interactions, while metabolic reprogramming intersects with inflammatory signalling to influence disease progression and treatment outcomes. This Research Topic also surfaces important unresolved questions. How might combinations of immunomodulatory strategies such as, Treg targeting, HIF-α modulation, or metabolic intervention, augment conventional therapies without inducing immunopathology? What molecular mechanisms underlie the epistatic interactions observed in murine models, and how do these translate to human genetic diversity? Can senescent T cell accumulation be prevented or reversed? Might metabolomic profiling enable early detection of treatment failure before clinical relapse? And how can macrophage-directed strategies be safely translated from bench to bedside? Addressing these questions will require integrative multi-omic approaches, encompassing genomics, transcriptomics, proteomics, and metabolomics, combined with spatial and single-cell technologies capable of resolving immune heterogeneity across tissues and time, validated across diverse parasite species, host backgrounds, and clinical contexts.

This Research Topic represents a meaningful step toward a systems-level understanding of leishmaniasis. By embracing the complexity of immune networks, host genetics, tissue context, and metabolic regulation, these studies deepen fundamental understanding while highlighting practical avenues for improved diagnostics, immunotherapies, and vaccines for a neglected tropical disease that continues to affect the world’s most vulnerable populations.

